# Development of electrophysiological and morphological properties of human embryonic stem cell-derived GABAergic interneurons at different times after transplantation into the mouse hippocampus

**DOI:** 10.1371/journal.pone.0237426

**Published:** 2020-08-19

**Authors:** Swechhya Shrestha, Nickesha C. Anderson, Laura B. Grabel, Janice R. Naegele, Gloster B. Aaron

**Affiliations:** 1 Department of Biology, Wesleyan University, Middletown, Connecticut, United States of America; 2 Program in Neuroscience and Behavior, Wesleyan University, Middletown, Connecticut, United States of America; National University of Ireland Galway, IRELAND

## Abstract

Transplantation of human embryonic stem cell (hESC)-derived neural progenitors is a potential treatment for neurological disorders, but relatively little is known about the time course for human neuron maturation after transplantation and the emergence of morphological and electrophysiological properties. To address this gap, we transplanted hESC-derived human GABAergic interneuron progenitors into the mouse hippocampus, and then characterized their electrophysiological properties and dendritic arborizations after transplantation by means of *ex vivo* whole-cell patch clamp recording, followed by biocytin staining, confocal imaging and neuron reconstruction software. We asked whether particular electrophysiological and morphological properties showed maturation-dependent changes after transplantation. We also investigated whether the emergence of particular electrophysiological properties were linked to increased complexity of the dendritic arbors. Human neurons were classified into five distinct neuronal types (Type I-V), ranging from immature to mature fast-spiking interneurons. Hierarchical clustering of the dendritic morphology and Sholl analyses suggested four morphologically distinct classes (Class A-D), ranging from simple/immature to highly complex. Incorporating all of our data regardless of neuronal classification, we investigated whether any electrophysiological and morphological features correlated with time post-transplantation. This analysis demonstrated that both dendritic arbors and electrophysiological properties matured after transplantation.

## Introduction

Temporal lobe epilepsy (TLE) accounts for approximately 60% of all seizure disorders, and is often acquired after traumatic injuries including stroke, ischemia, tumor, fever or infection [1, 2]. The progressive loss or dysfunction of subsets of hippocampal GABAergic interneurons is one of the pathological hallmarks of both human and rodent TLE [3–11]. Managing the most challenging epileptic disorders–those that are drug-resistant, multifocal, and non-resectable–will require novel treatments, such as stem cell-based therapy. Reliable protocols are now available for making human embryonic stem cell (hESC)-derived GABAergic interneurons [12, 13]. Following transplantation into rodent brains, hESC-derived GABAergic interneurons appear to undergo a protracted process of differentiation that can last many months before they acquire mature electrophysiological properties [13–16]. Several studies established a correlation between the emergence of electrophysiological properties and morphological development of endogenous neurons [17–23], but whether hESC-derived GABAergic neurons transplanted into adult mouse brains attain functional and structural maturity is not well understood.

To address this gap, we investigated whether the electrophysiological and morphological properties of hESC-derived human neurons show maturation-dependent changes after transplantation. We derived MGE-like ventral forebrain neural progenitors from the hESCs and transplanted them into the hippocampus of immunodeficient NSG mice. After allowing ~16–24 weeks for maturation of the transplanted cells, we carried out whole-cell patch clamp recordings in hippocampal slices to assess the electrophysiological properties of the transplanted cells, and filled them with biocytin. We performed confocal microscopy and reconstructed the dendritic arbors with 3-dimensional (3D) computer-based software. Based on firing responses to current injections, five electrophysiological types (Type I-V) of transplanted hESC-derived interneurons were described in our previous study [14]. In the present study, we extended these analyses by performing hierarchical clustering on the basis of dendritic arbor morphology. This analytical approach suggested that the cells could be subdivided into four morphologically unique cell classes (Class A-D) and further analyses suggested a link between these classes and electrophysiological types. Overall, we assessed the development of electrophysiological maturity and dendritic growth of transplanted human neurons over time in the mouse brain. These findings provide a foundation for future studies assessing the time course for feasibility of transplanting human interneurons for therapeutic purposes.

## Materials and methods

### Neural induction and differentiation

The cell lines used for transplantation were hES3 NKX2.1: GFP (gift from Dr. Stewart A. Anderson and Dr. Andrew Elefanty) and a modified variant of this line designated hES3 NKX2.1: GFP/ubi: mCherry [14]. The neural induction and interneuron differentiation protocols were as described previously [14, 24]. Briefly, the hESCs were differentiated in LDN (BMP antagonist), Sonic hedgehog (SHH) and the SHH agonist Purmorphomine (Pur) for approximately 18 days, then frozen in liquid nitrogen for storage until they were thawed and dissociated for transplantation.

### Animals

Animal usage protocols were approved by the Wesleyan University Animal Care and Use Committee and were conducted according to regulations established by the National Institute of Health for the care and use of laboratory animals. The data in this study were obtained from 26 adult male and female immunodeficient mice (NOD.Cg-Prkdc SCID I12rgtm 1Wjl/SzJ; NSG, Jackson Labs). The mice were housed in pairs in self-ventilating cage racks on a 12-hour light/dark cycle and provided with food and water ad libitum.

### Transplantation of hESC-derived interneuron progenitors

We transplanted hESC-derived interneuron progenitors into multiple sites in the dorsal hippocampus, as described previously [14]. Briefly, ~6 weeks old NSG mice were anesthetized with isoflurane gas and stereotaxic surgery was performed using a Quintessential Injection System (Stoelting Co.). Approximately 50,000 differentiated hESC-derived neural progenitors in 0.5 μl of media were transplanted bilaterally into two sites in the hippocampus at the coordinates (AP-2.4, ML-2.2, DV-2.2, 2.0).

### Slice preparation and electrophysiology

Patch-clamp electrophysiological recordings were conducted, as described previously [14]. Briefly, ~16–24 weeks after transplantation, we deeply anesthetized the mouse with ketamine hydrochloride (120 mg/kg ketamine; i.p.; Ketaset, Zoetis), xylazine (10 mg/kg; i.p.; Anased, Lloyd Laboratories) and euthanized by decapitation. The brain was quickly removed and placed into ice-cold high sucrose artificial cerebrospinal fluid (ACSF) (27.07 mM NaHCO_3_, 1.5 mM NaH_2_PO_4_, 1 mM CaCl_2_, 3 mM MgSO_4_, 2.5 mM KCl, 222.14 mM sucrose). Thick horizontal sections (350 μm) were cut on a vibratome (Leica, VT1000S, Leica Microsystems Inc., Bannockburn, IL, U.S.A.) and maintained in a chamber while continuously perfusing them with oxygenated ACSF (37°C; 125 mM NaCl, 1 mM CaCl2, 3 mM MgSO4, 1.25 mM NaH2PO4, 25 mM NaHCO3, 2.5 mM KCl, 25 mM glucose, 3 mM myo-inositol, 2 mM Na-pyruvate, and 0.4 mM ascorbic acid).

Based on expression of Green Fluorescent Protein (GFP) or Red Fluorescent Protein (RFP)-expressing cells, we identified the transplanted human GABAergic interneurons and then carried out whole-cell current clamp recordings in these cells under infrared-differential interference contrast (IR-DIC). Voltage changes were measured in response to graded positive and negative current injections (500 ms durations). Whole-cell voltage clamp recordings were performed in identified transplanted cells to measure spontaneous inhibitory postsynaptic currents (IPSCs) and excitatory postsynaptic currents (EPSCs) at -10mv and +70mV respectively. Analog signals were digitized at a sampling rate of 40 kHz (using ITC-18) and captured with IGOR software (Wavemetrics). To link the recordings of the neurons to their morphology, we filled the cells with biocytin contained in the intracellular recording solution in the patch pipettes (130mM potassium methansulfonate, 10 mM HEPES, 5 mM NaCl, 2.5 Mg-ATP, 0.3 NA-GTP, 11 mM Biocytin).

### Data analysis of electrophysiological recordings and morphologies

#### Electrophysiology

We analyzed current clamp recordings in IGOR software to calculate the following electrophysiological characteristics: action potential (AP) firing rate, AP half-width, adaptation, repolarization rate, accommodation and input resistance, as described previously [14]. Based on their AP characteristics, the recorded cells were divided into five types; Type I (highly adapting), Type II (fast-spiking like), Type III (bursting), Type IV (accommodating), and Type V (non-accommodating).

#### Immunohistochemistry

Subsequent to the electrophysiology recordings, the slices were fixed overnight in 4% paraformaldehyde (Electron Microscopy Sciences) in 0.1 M phosphate buffer (pH 7.4). The transplanted cells were identified in the slices by immunofluorescent staining for human nuclear antigen (mouse anti-HuNu, 1:1000, Chemicon) or the expression of RFP (rabbit anti-RFP 1:1000, Rockland,). Biocytin staining was performed by incubating the slices in Streptavidin 647 (1:1000, Life Technologies) [14].

#### Confocal imaging

Following electrophysiological recordings, fixation and immunostaining of hippocampal slices, confocal imaging was performed, as described [14]. Confocal microscopic z-stacks were made of the recorded and biocytin-stained neurons with a 25x objective, a 0.3 μm step size (Zeiss LSM 510) and a resolution of 2048 by 2048 pixels. For larger images, multiple z-stacks were obtained.

#### 3D neuronal reconstructions of biocytin-filled cells

To analyze the dendritic morphology of biocytin-filled human neurons, we imported the confocal images into IMARIS (version 9.2, Bitplane) neuronal reconstruction software. Larger neurons that exceeded the imaging volume required multiple confocal z-stacks, and these were stitched using 3D stitching software (XUV Stitch). The somas of neurons were reconstructed using the surface tracer tool and dendritic arbors were traced using the filament tracer tool. The morphometric features quantified in IMARIS software included: soma volume, total dendritic length, and number of terminal points. The tracings from the center of the soma to the beginning of primary dendrites were excluded while calculating total dendritic lengths. To compare dendrite arbors, we performed Sholl analyses with radii separation at 10 μm intervals and quantified the number of dendritic branch crossings. The maximum dendritic radius and total dendritic intersections were determined with Sholl analyses.

#### Morphological categorization of reconstructed neurons

Hierarchical clustering analyses were performed in R statistical software, taking into account morphometric features. These were: the number of primary dendrites, the total dendritic length, the number of terminal points, the maximum radial extent, and the maximum intersections. Based on these features, we were able to identify four morphologically unique morphological classes (A-D).

### Statistical analyses

Spearman correlation coefficients were calculated (using GraphPad Prism-7) to establish a relationship between various electrophysiological properties and morphological characteristics versus the duration of transplants. An ANOVA with Tukey’s post hoc analysis (using GraphPad Prism-7) was used to make comparisons between four morphologically unique classes and between the firing rates of five previously described five electrophysiological types [14].

## Results

We previously investigated the functional effects of transplanting hESC-derived interneuron progenitors on cognition and seizures in the mouse pilocarpine model of TLE. At the time of transplantation, the GABAergic progenitors were greater than 90% Nestin and Musachi positive [14]. In addition, approximately 55% of the cells continued to express the interneuron progenitor marker NKX2.1 [14]. By 16 weeks post-transplantation, ~90% of the transplanted cells became mature neurons with cells expressing the inhibitory neurotransmitter GABA as week as interneuron subtypes SST, CB and CR [14]. While there was significant maturation of the cells post-transplant, a small percentage of transplanted cells remained as neural progenitors, which led to the development of tumor-like clusters in some animals [14]. Overall, we observed that many of the transplanted cells integrated into the hippocampus and acquired mature molecular, electrophysiological and morphological properties. We also found some rescue of spatial memory deficits in mice with transplants compared to controls, although we did not find significant reductions in the incidence of spontaneous recurrent seizures [14].

**Fig 1:**
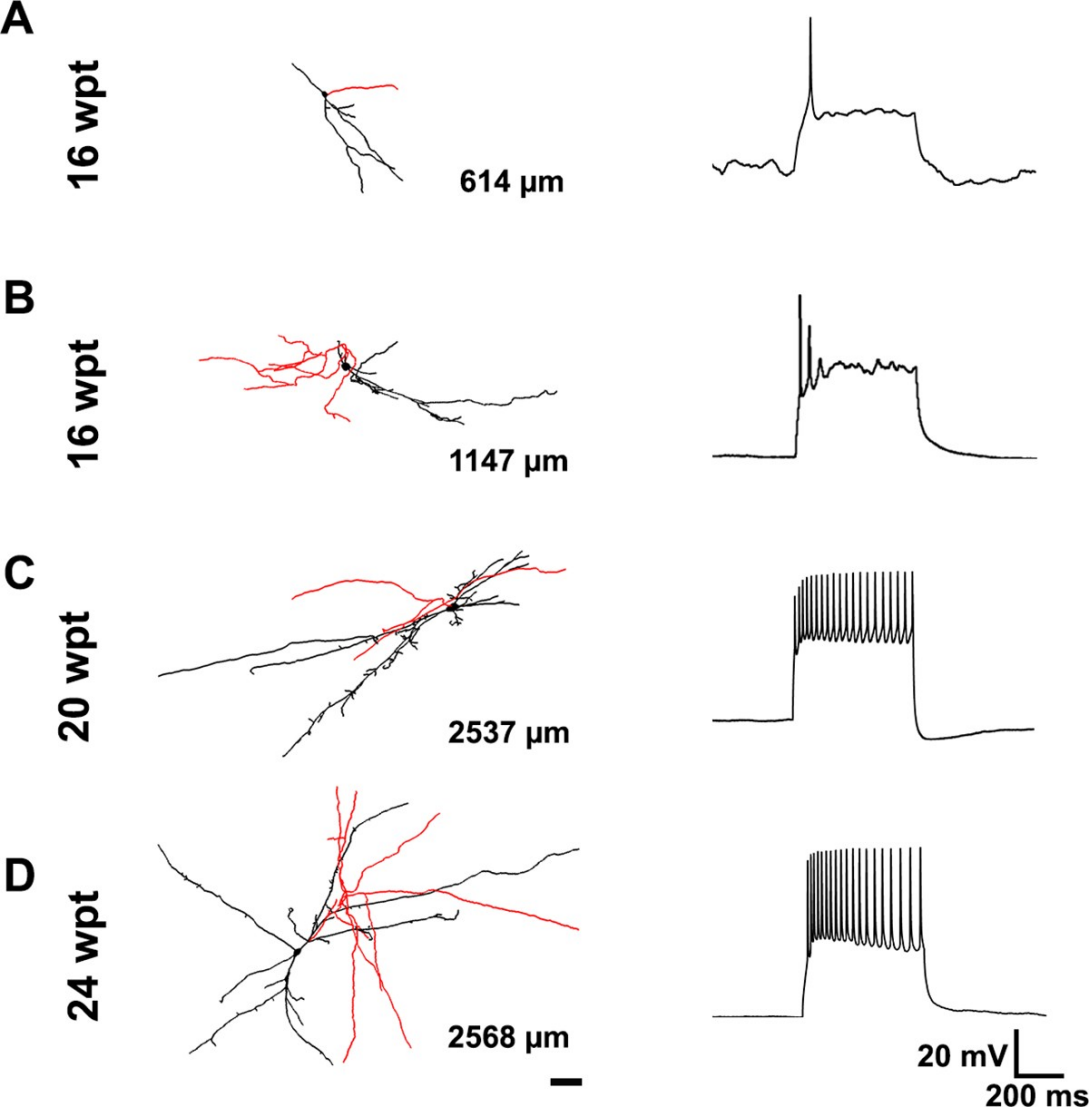
Morphology and electrophysiology of transplanted cells at different times post-transplantation. Representative examples of transplanted cells with their respective morphology and electrophysiology at 16 wpt (A), 16wpt (B), 20 wpt (C) and 24 wpt (D). The total dendritic lengths in A, B, C and D equal 614μm, 1147μm, 2537μm and 2568μm, respectively. Putative dendrites and putative axons are shown in black and red, respectively. Scale bar equals 50μm. wpt: weeks post-transplant.

We carried out the present study to investigate the development of electrophysiological and morphological properties at different time points after transplantation in the mouse hippocampus. To delineate the timeline for the maturation of transplanted human neurons, we analyzed dendritic arbor maturation. In general, the cells with the shortest post-transplantation durations exhibited simpler dendritic arbors and immature electrophysiological properties (Fig 1A and 1B). In contrast, cells with longer post-transplantation durations exhibited more elaborate distal dendritic arbors and more mature electrophysiological properties (Fig 1C and 1D).

**Fig 2:**
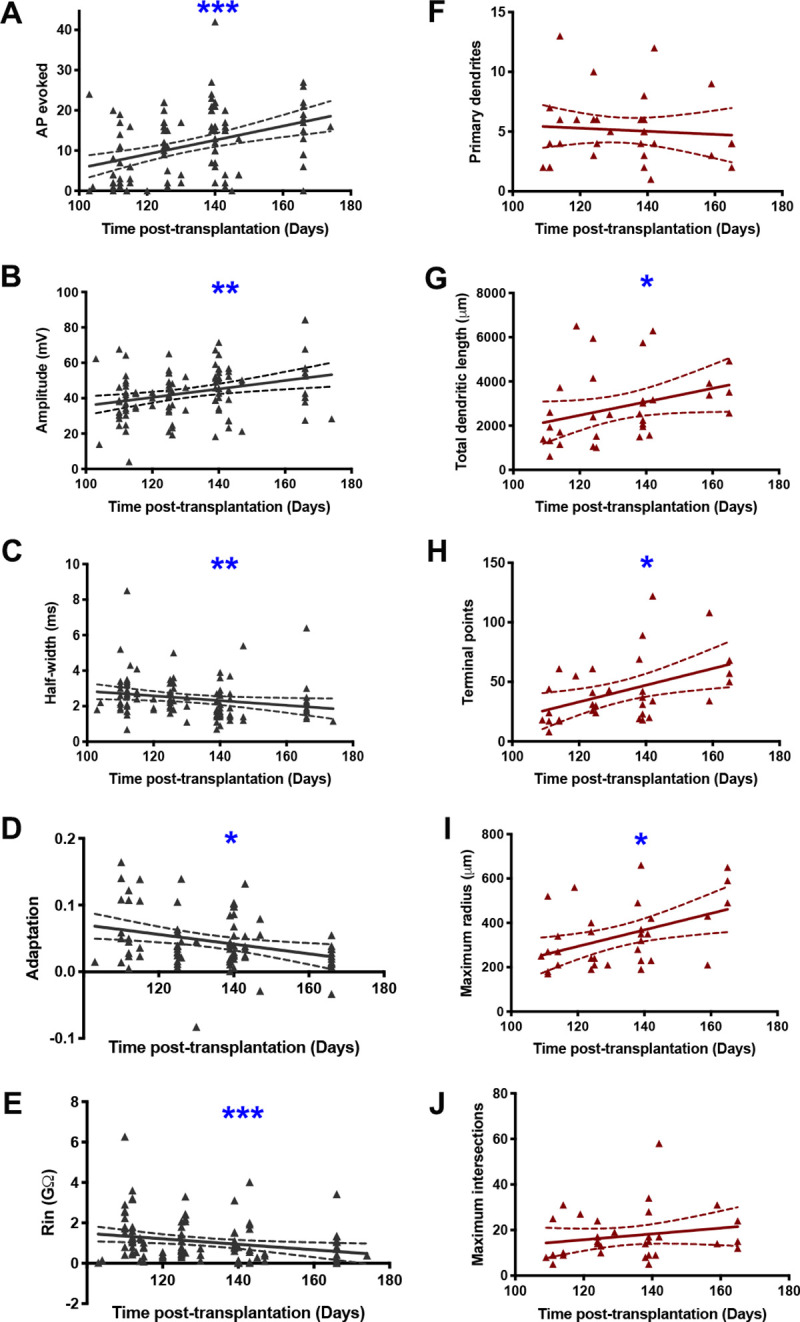
The development of electrophysiological and morphological properties over the time post-transplantation. (A) Number of APs evoked (neurons successfully whole-cell patch clamped with multiple current injections, n = 107). (B) Amplitude of APs (neurons that evoked at least one AP, n = 104). (C) AP half-width (n = 104 neurons). (D) AP adaptation (neurons that evoked at least 4 APs, n = 80) (E) Input resistance (all neurons successfully whole-cell patch-clamped, n = 110). For figures *F–J*, only neurons with complete dendritic reconstructions are eligible, n = 32 neurons for each. (F) Number of primary dendrites. (G) Total dendritic length. (H) Number of terminal points. (I) Maximum radius, Sholl analysis. (J) Maximum number of intersections, Sholl analysis. The straight line represents the line of best fit with 95% confidence interval. Spearman’s correlation coefficient shows the relationship of various electrophysiological and morphological properties over the duration of transplants (*p< 0.033, **p<0.02, ***p<0.001).

Electrophysiological properties of transplanted cells that we measured included maximum number of action potentials evoked during a 500ms current injection, action potential amplitude, and action potential half-width. Significant positive correlations were found between the number of action potentials evoked and time post-transplantation, as well as action potential amplitudes and time post-transplantation. The action potential half-widths, adaptation and input resistance were negatively correlated with the time post-transplantation (Fig 2A–2E). These findings indicate maturation of electrophysiological properties over time.

Dendritic morphology was studied with IMARIS 9.2 (Bitplane) by making 3D computer-based reconstructions of 75 biocytin-filled neurons. Of these, 32 were completely reconstructed and their morphological features were quantified, including: soma volume; primary dendrites; total dendritic length; dendritic terminal points and dendritic complexity. We examined all of these properties to determine whether any showed significant changes over the time post-transplantation. We found significant positive correlations between the time post-transplantation and all the following measurements: total dendritic length; terminal points; and maximum radii (Fig 2G–2I). However, there were no correlation between the number of primary dendrites and the number of maximum intersections over time, suggesting increases in the growth of transplants distally over time but not proximally (Fig 2F–2J). Together these findings suggest that the length of time after transplantation is a good predictor for assessing electrophysiological and morphological maturity of transplanted cells.

**Fig 3:**
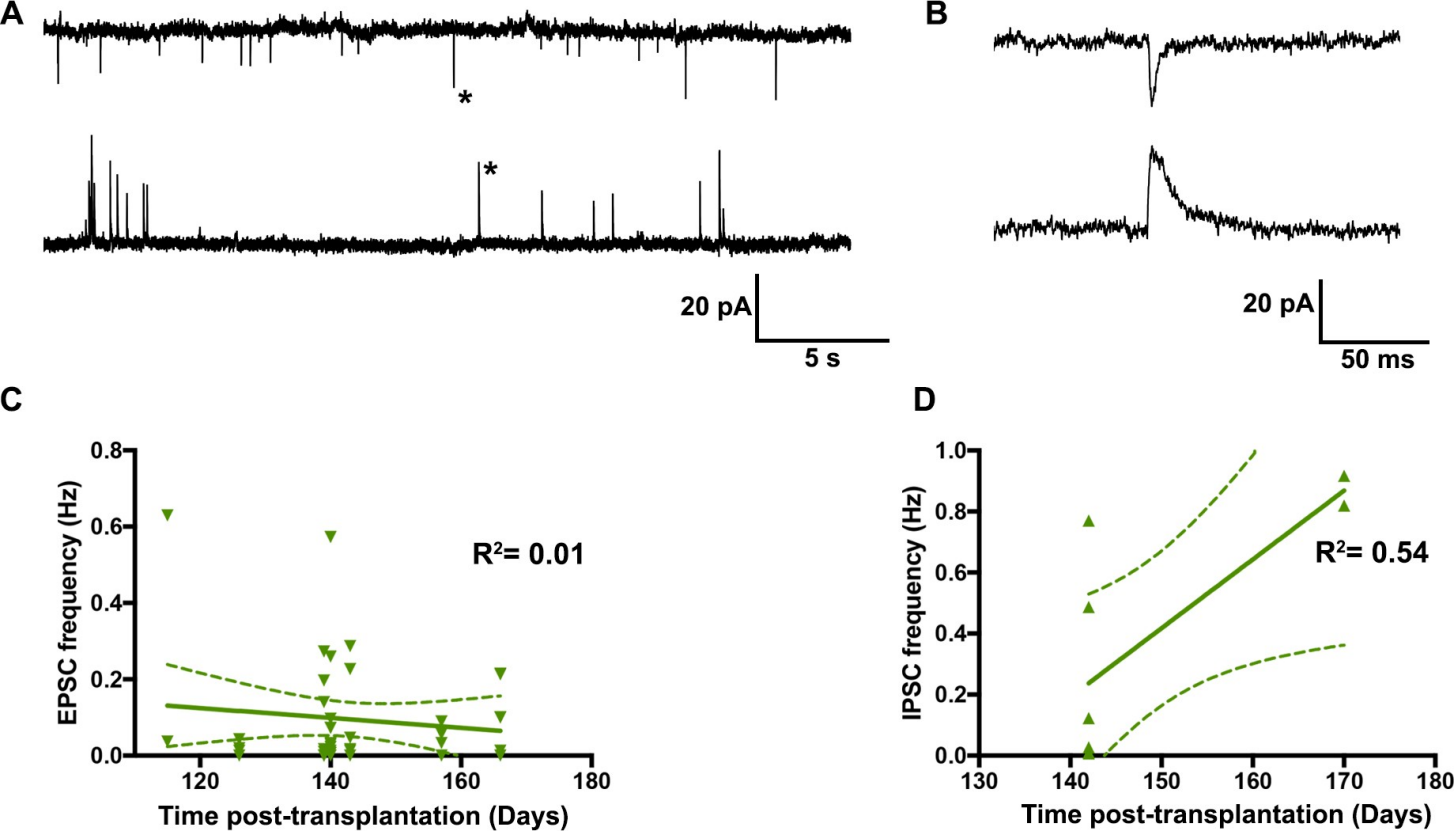
The development of host synaptic inputs over the time post-transplantation. (A) Voltage clamp recordings of transplanted neurons at -70mV (top, EPSCs) and +10mV (bottom, IPSCs). (B) Enlarged view of EPSC and IPSC indicated by asterisks. (C and D) Graphs show no correlation between EPSC frequency (C, n = 42 neurons) and IPSC frequency (D, n = 8 neurons) over the time post-transplantation.

We further investigated whether the frequency of synaptic inputs changes over the time post-transplantation. While we recorded 34/42 transplanted cells that received EPSCs and 8/8 transplanted cells that received IPSCs, we did not find any significant correlation in the frequency of synaptic inputs over the duration of transplants (Fig 3).

**Fig 4:**
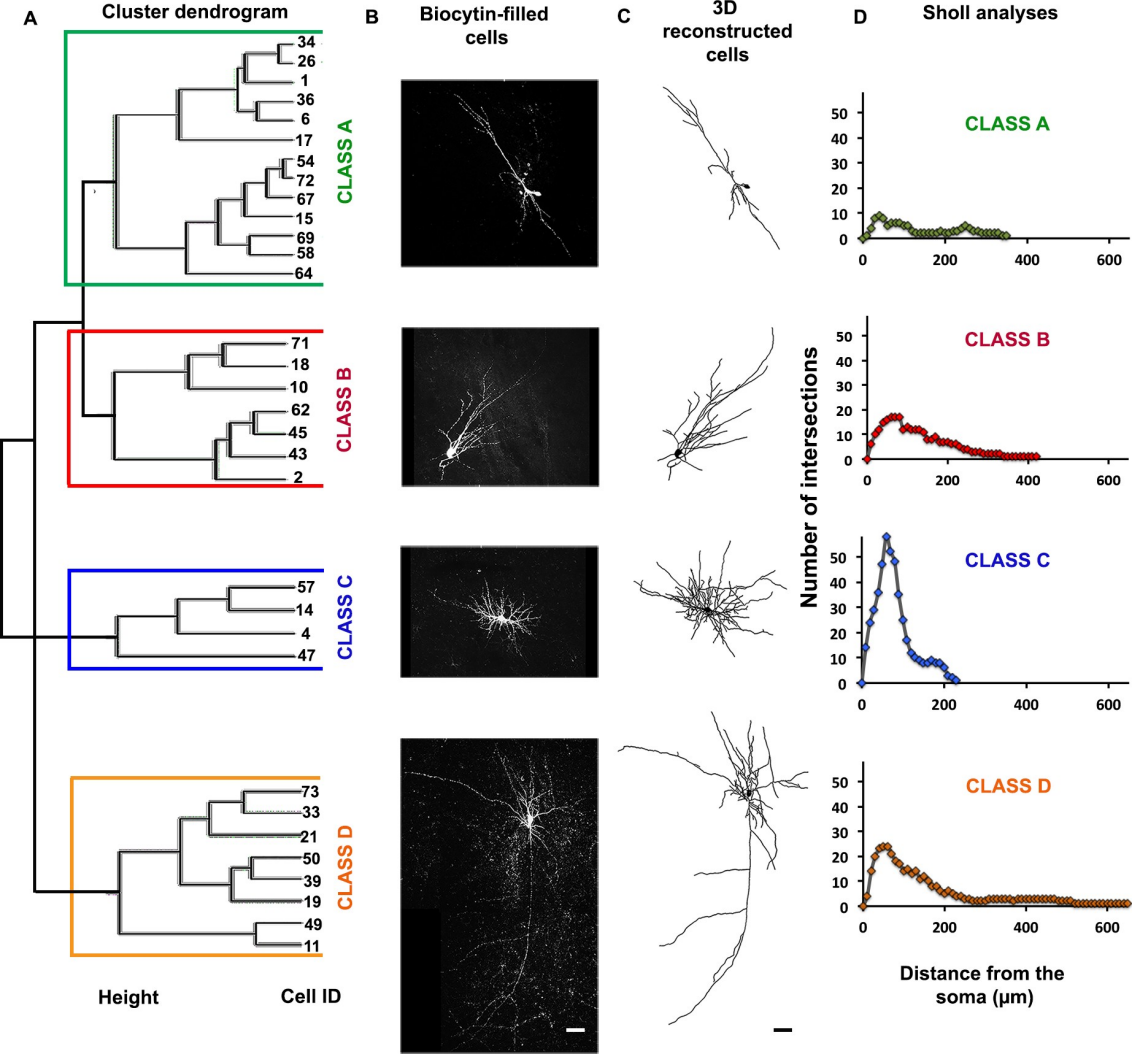
Four unique morphological classes. (A) Cluster dendrogram of four unique morphological classes were created using hierarchical clustering analyses: Class A (green bracket), Class B (red bracket), Class C (blue bracket), and Class D (yellow bracket) Y-axis: height and X-axis: Cell ID. (B) Representative examples of biocytin-filled neurons from each morphological class. (C) The neuronal reconstructions of biocytin-filled neurons. (D) Sholl analyses of neurons displayed in *C*. Soma and dendrites are shown in black. Class A (n = 13), Class B (n = 7), Class C (n = 4) and Class D (n = 8). Only neurons with complete neuronal reconstructions included (total for all classes combined, n = 32). Scale bars represent 50μm.

**Fig 5:**
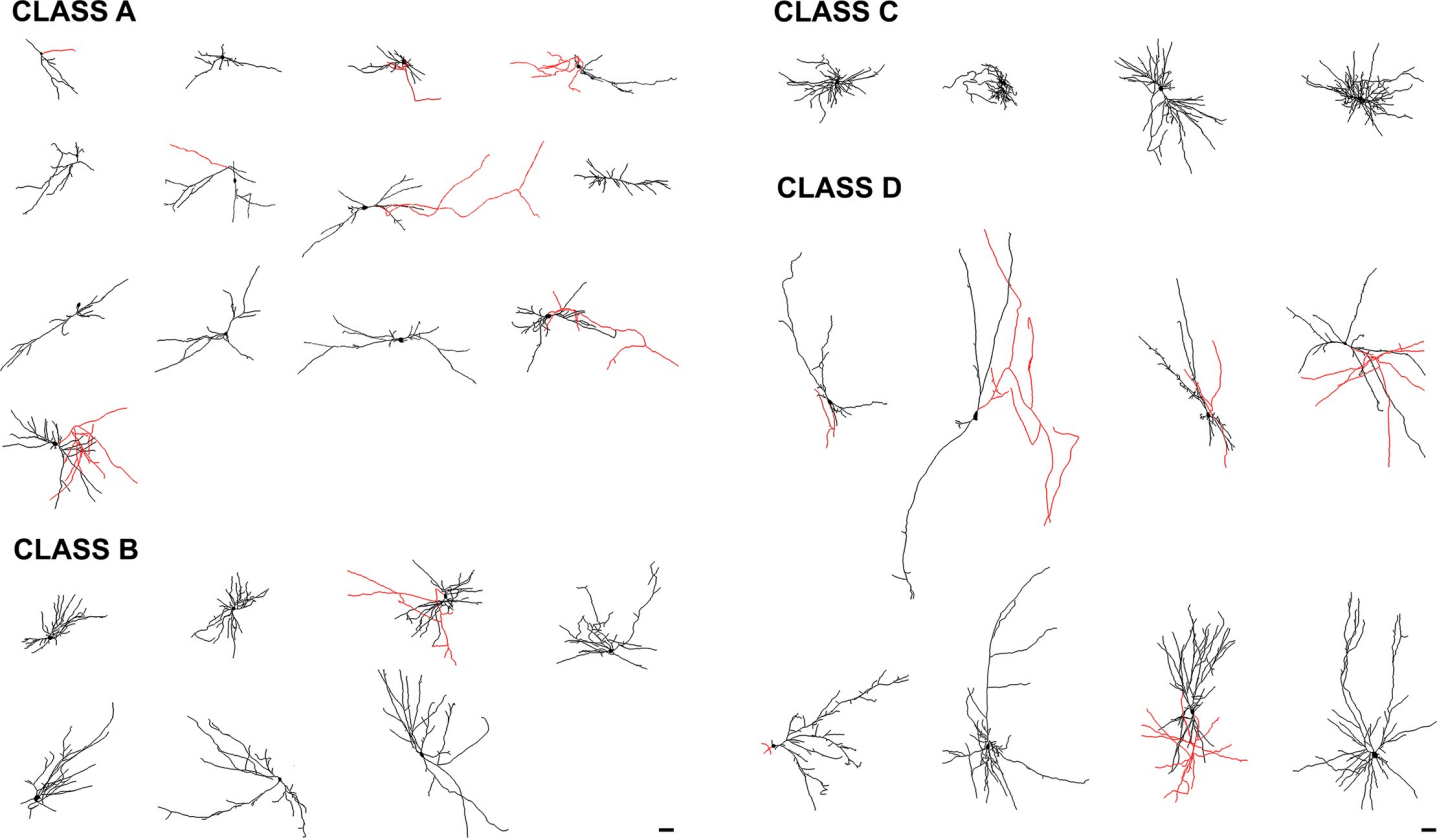
Gallery of transplanted human interneurons with complete reconstructions of dendritic arbors. The human neurons exhibited a range of morphological phenotypes and were grouped based on dendritic arbor morphology and complexity. Class A contained cells with simple dendritic arbors. Class B cells had intermediate dendritic arbor complexity. Class C cells had compact, tortuous and bushy dendritic arbors. Class D cells were large, with complex dendritic arbors. Somas and dendrites are shown in black and putative axons are in red. N equals: Class A (n = 13), Class B (n = 7), Class C (n = 4) and Class D (n = 8). Scale bars equal 50μm.

Formerly, patch-clamp analyses suggested four distinctive firing patterns: highly adapting (Type I), fast spiking (Type II), bursting (Type III), accommodating (Type IV) and non-accommodating (Type V) [14]. To examine whether the electrophysiological maturation of the transplanted neurons were associated with progressive stages of dendritic arbor maturation, we performed hierarchical clustering analyses (using R) that incorporated various dendritic measurements, allowing us to identify four morphologically distinct classes: Class A, Class B, Class C, and Class D (Figs 4 and 5). Analyses of the biocytin-filled cells revealed an array of different dendritic arbor morphologies, with the majority resembling stellate or multipolar cells (n = 27), and a few that were unipolar (n = 2) or bipolar (n = 3) (Fig 5). In addition, the cells had diverse somatic morphologies including: fusiform (n = 15), round (n = 6), pyramidal (n = 5), polygonal (n = 3) or curved (n = 3) (Fig 5).

**Fig 6:**
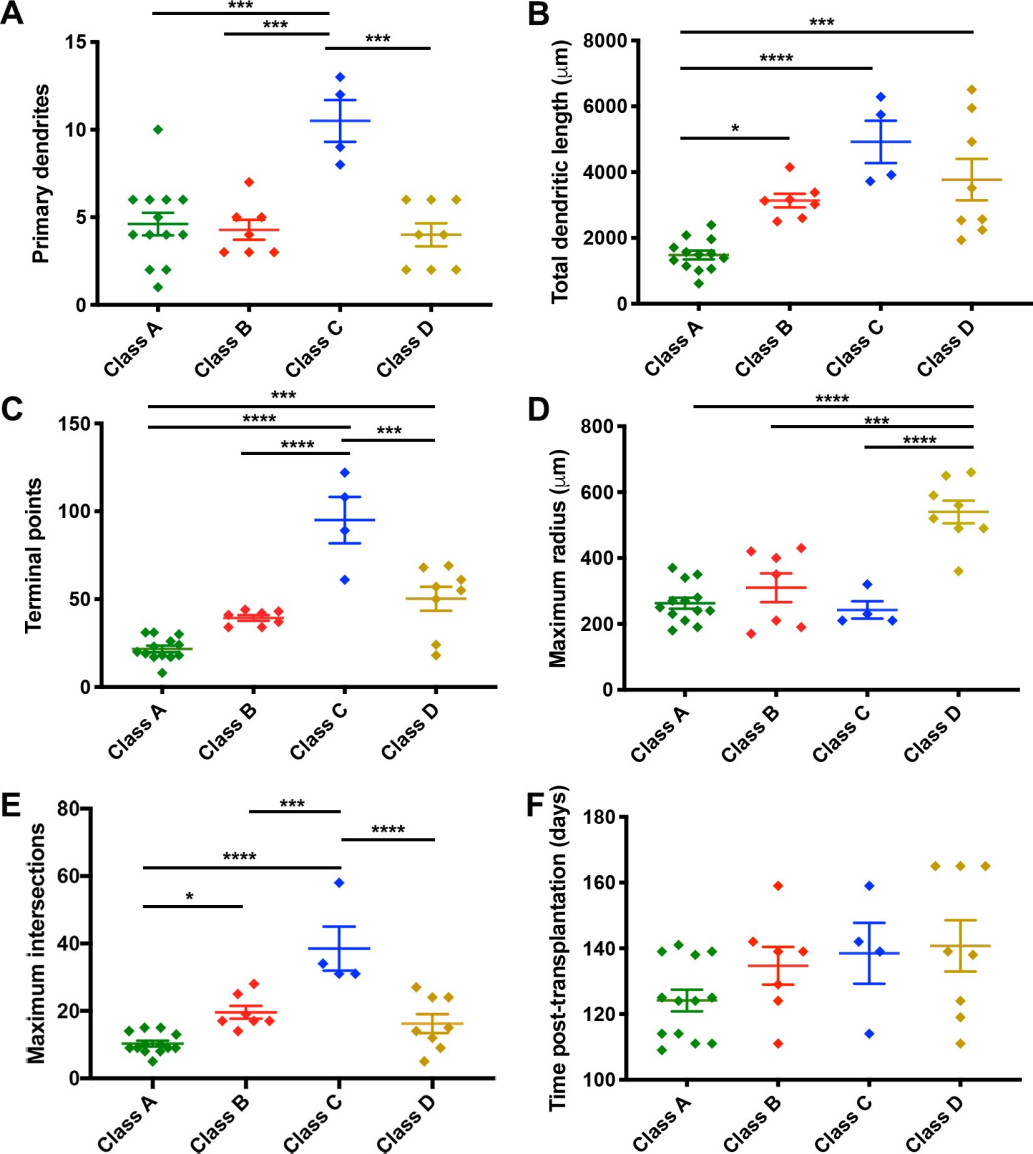
Graphs displaying morphometric features of four morphological clusters. The graphs show the difference in the following: (A) number of primary dendrites; (B) total dendritic length; (C) number of terminal points; (D) maximum radius; (E) maximum intersections; (F) and the number of days post-transplantation between four morphological clusters. Class A (n = 13), Class B (n = 7), Class C (n = 4), and Class D (n = 8). Results are represented as mean ± SEM (ANOVA with Tukey’s multiple comparison test, ****p < .0001, *** < .001, ***p<0.001, ** p<0.01, * p<0.05.

Class A had the lowest number of primary dendrites, terminal points, maximum intersections, and the shortest total dendritic lengths. The simple immature appearance of these cells suggested that they were still in the early stages of differentiation. Class B cells also had significantly fewer primary dendrites and terminal points compared to Class C cells but their total dendrites were significantly longer and the maximum intersections were higher than Class A cells suggesting further maturation. Our interpretation is that the neurons in this group were also immature but developing more extensive dendritic arbors. Class C cells had significantly more primary dendrites, terminal points, and maximum intersections than Class A, B and D cells, however, the dendritic arbors were significantly smaller than Class D cells. The cells in this group therefore appear to exhibit mature, compact dendritic arbors, with a high density of dendritic branches. Class D cells had a significantly larger maximal radial extent than Classes A- C and significantly larger dendritic arbors and higher number of terminal branch points than Class A cells. Thus, the cells within Class D were the largest and most complex suggesting that these cells comprise a mature neuronal Class (Fig 6 and Table 1). Despite the significant differences in morphometric properties between the classes, we reported no significant differences in the time post-transplantation between the classes.

**Table 1:** Properties used to define morphologically distinct cell classes.

	Class A	Class B	Class C	Class D	P < 0.5
**# Primary Dendrites**	4.6 ± 0.65	4.29 ± 0.57	10.5 ± 1.19	4.0 ± 0.65	A-B, A-C, A-D
**Total Dendritic Length (μm)**	1484 ± 33.60	3136 ± 206.30	4920 ± 646.60	3773 ± 631	A-C, C-D, B-C
**# Terminal Points**	21.69 ± 1.86	39.29 ± 1.60	95 ± 13.20	50.25 ± 6.79	A-C, A-D, A-B, C-D
**Maximum Radius (μm)**	263.10 ± 16.54	310 ± 43.70	285.50 ± 30.16	540 ± 34.64	A-D, B-D, C-D
**Maximum Intersections**	10.31 ± 0.84	19.57 ± 1.90	38.50 ± 6.54	16.25 ± 2.80	A-C, A-D, A-B, B-C
**Time post-transplantation (Days)**	138.50 ± 9.28	134.70 ± 5.75	124.20 ± 3.30	140.80 ± 7.81	ns

Table 1 displays the mean and the standard error of mean of five morphometric variables that were incorporated in hierarchical clustering analyses to identify four morphologically unique clusters as well as the time post-transplantation of each morphological cluster. N = 32 neurons.

**Fig 7:**
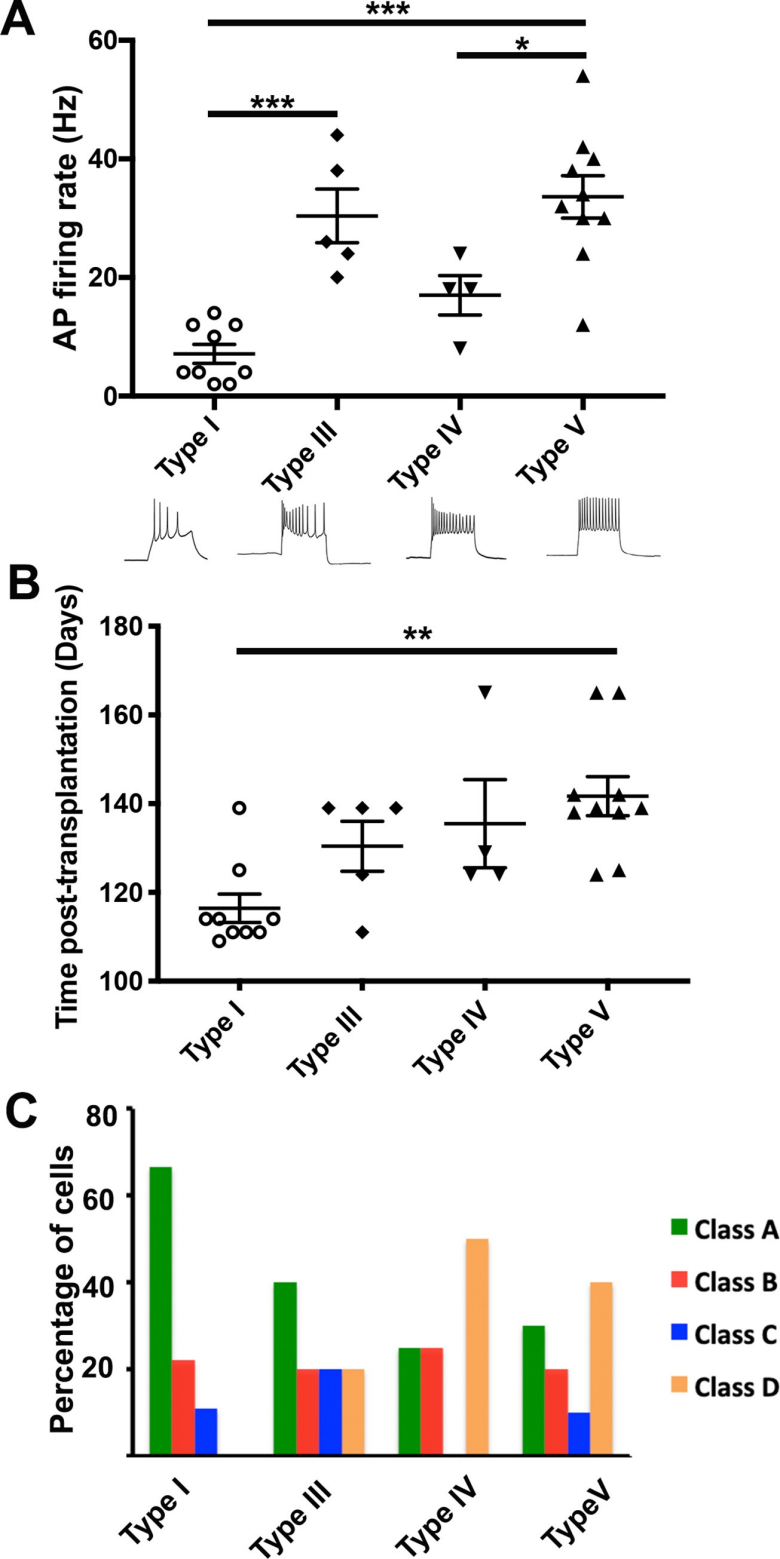
Electrophysiological and morphological relation of transplanted cells. (A) The differences in the AP firing rate between subsets of different electrophysiological types (top) and representative examples of firing patterns from each type (below). These electrophysiological types are previously published [14]. (B) The differences in the duration of transplants between different electrophysiological types. (C) The percentage of cells from different morphological classes within each electrophysiological type. Type I (n = 9), Type III (n = 5), Type IV (n = 4), and Type V (n = 10). Only neurons with complete neuronal reconstructions and at least 4 APs during a 500 ms current injection included (n = 28). Results are represented as mean ± SEM (ANOVA with Tukey’s multiple comparison test, ****p<. 0001, *** < .001, * p<0.05.

**Fig 8:**
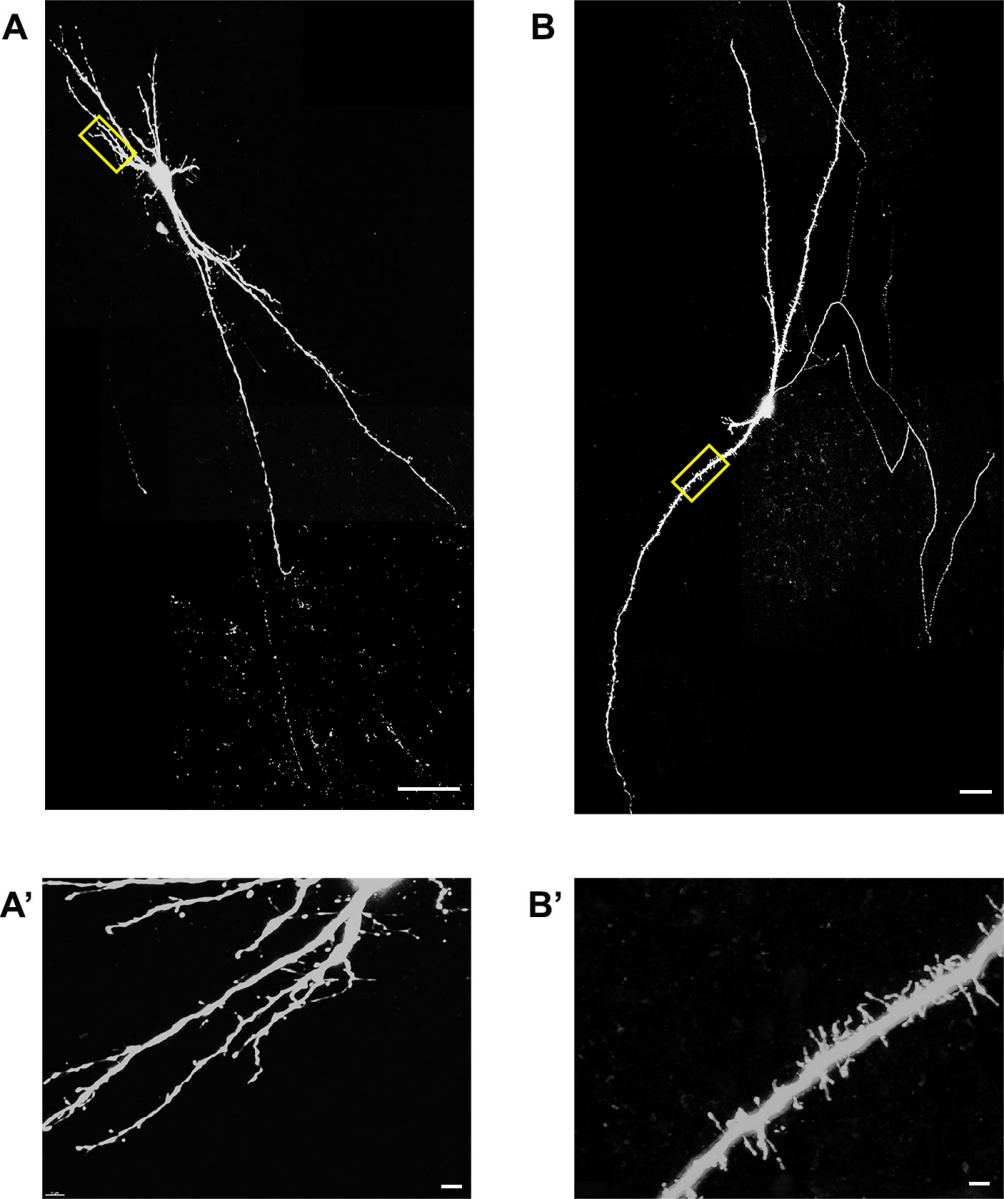
Representative examples of spiny neurons. Two examples of biocytin-filled neurons (A-B), scale bars represent 50μm. Zoomed-in views of insets, showing dendritic spines (A’-B’). Scale bars equal 5μm.

Taking into account only the subset of transplanted neurons with complete dendritic arbors, we found significantly lower AP firing rates of Type I cells, compared to Type V (Fig 7A). Interestingly, Type I cells consisted of transplanted human interneurons that were significantly younger (116 ± 3.2 days) than the Type V cells (141.7 ± 4.4 days) (Fig 7B). Consistent with our hypothesis that Type I cells were immature, we found that 66.7% of Type I cells fell into Class A; these cells had the least mature and simplest dendritic arbors. Likewise, Type V cells with mature firing patterns comprised about 40% of the Class D cells with complex dendritic morphologies (Fig 7C). Furthermore, of the 6 spiny neurons, 4 were assigned to the Class D cluster (Fig 8) consistent with this group’s more mature electrophysiological and morphological properties.

## Discussion

Development of dendritic arbor morphology and electrophysiological properties are important aspects of neuronal maturation that ultimately determine neuronal function and connectivity. Previously, we had quantified various electrophysiological properties of transplanted cells and identified five electrophysiological types based on firing patterns. These were: Type I, highly adapting; Type II, fast-spiking; Type III, bursting; Type IV, accommodating and Type V, non-accommodating [14]. Electrophysiological based assessments are a well-established method for distinguishing mature interneuron subtypes. However, previous studies found that neural progenitors differentiate over a protracted period after transplantation [13, 14, 16]. Here, we carried out detailed computer-based 3D reconstructions of the cells to quantify their morphological features. Then, we investigated the development of electrophysiological and morphological properties of transplanted cells at different times after transplantation. We also evaluated whether our electrophysiological types correlated with morphological classes we formed.

Our data suggest the on-going maturation of human transplants in the mouse brain. Specifically, we found that transplanted human interneurons develop more elaborate dendritic arbors and mature electrophysiological properties over ~14–24 weeks post-transplantation into the mouse hippocampus. Despite the maturity of electrophysiological and morphological properties, we did not find a correlation between the synaptic inputs and the time post-transplantation. We might not have seen this relationship because most of the excitatory and inhibitory synaptic inputs were recorded from a narrow duration of time, especially with regards to IPSCs. With regards to EPSCs, it’s also possible that we might have failed to capture synaptic inputs arising from distal dendrites that appeared to be the area where growth was measured morphologically, due to space clamp issues that can prevent effective voltage clamping of those most distal sites.

We reported significant differences in the time post-transplantation between the electrophysiological Type I and V, implying that these types to be different maturational states rather than unique interneuron subtypes. In contrast, we did not find any significant differences in the time-post transplantation among any morphological classes we formed, although we did show positive maturation of some morphological properties, such as the growth of distal dendrites over time (Fig 2). This conclusion suggests that simple dendritic arborization does not always associate with an early stage of differentiation and vice versa. In other words, it is possible that inherent morphological classes are more distinct than the distinctions that would develop over time. For example, it is likely that a fully mature neuron in one category may have shorter dendrites than a less developed neuron in a different category. Many research groups, including the Petilla interneuron nomenclature group, have shown examples of dendritic morphologies of mature interneurons from human cerebral cortex that are both simple and elaborate. Ideally, we would have a much larger sample of neurons so that we could examine development within each class, but this will have to wait for a future study.

While we did not find a strict relationship between the electrophysiological types and morphological clusters, we showed some association between electrophysiological types and morphological classes, primarily between Type I and Class A. In our sample of transplanted hESC-derived neurons, 66.7% had simple dendritic morphologies (Class A) and immature firing patterns (Type I) while 40% had complex dendritic arbors (Class D) and mature firing patterns (Type V). We found 4 out of 6 spiny neurons belonged in Class D, consistent with a previous report showing that the spiny dendrites can indicate electrophysiological and morphological maturity in some neurons [25]. This result is consistent with prior work in rat frontal cortex showing dendritic arbor morphology was correlated to the electrophysiology of fast-spiking (FS) and late-spiking (LS) interneurons although not in regular-spiking (RS) and burst-spiking (BS) interneurons [26]. The dendritic arbors of different kinds of interneurons include multipolar, bipolar, or bitufted, depending on what layer they reside in [27, 28].

We noted similarities between the dendritic morphologies of these hESC-derived GABAergic interneurons and drawings by Santiago Ramon y Cajal from Golgi-stained human GABAergic interneurons [29]. For example, many of our cells show a remarkable resemblance to human neurogliaform cells, basket cells and bipolar cells. Also, morphologies and physiologies of our transplanted cells look comparable to morphologies and physiologies of adult human GABAergic interneurons described in The Allen Human Brain Reference Atlas http://celltypes.brain-map.org/, maturing GABAergic interneurons derived from human pluripotent stem cells [13] and endogenous GABAergic interneurons from rodents [26, 28, 30–32]. Many research groups have combined dendritic and axonal morphologies along with their physiologies to distinguish GABAergic interneuron subtypes. In more recent study, morphological, electrophysiological and transcriptomic evidences were collectively used to study GABAergic neurons [33]. In the present study, we were able to identify axonal arbors in a small subset of reconstructions based on three overlapping criteria: first, a single process coming out of the soma or primary dendrite; second, thinner and non-tapering process; and last, the absence of spines. However, the axons were not considered while forming our morphological clusters, since most axonal arbors extended beyond the slice thickness used for these studies.

Based on previous findings, the transplanted cells comprise a molecularly heterogeneous group of neurons and by 16–24 weeks post-transplant, most expressed markers for interneuron subtypes such as somatostatin (SST), calbindin (CB), calretinin (CR) and parvalbumin (PV) [13, 14, 16]. Future work looking at the development and maturation of pluripotent stem cell (PSC) derived transplanted neurons should take into account single cell RNA sequencing and single-cell qRT-PCR analyses of gene expression to further identify and correlate GABAergic neuron subtypes with unique gene expression, distinctive dendritic arbor morphology, and defining electrophysiological properties.

Together with previous work showing synaptic and functional maturation of hESC-derived transplanted neurons, our data highlight the ability of human neurons to develop complex dendritic morphologies and exhibit mature electrophysiology after transplantation into the adult mouse hippocampus. Additionally, despite considerable heterogeneity in cell morphological and electrophysiological properties, we demonstrate here that the time following transplantation is a good predictor for the emergence of both dendritic complexity and electrophysiological maturity in human interneurons. We further established a link between our electrophysiological types and morphological classes. These findings provide a foundation for assessing structural and functional maturation of hESC-derived transplanted cells in mouse models of neurological disease that can be used in conjunction with gene expression studies. This work may also be helpful for predicting when behavioral tests for functional improvements would be most informative after transplantation.

## Supporting information

S1 Data (XLS)

S2 Data (XLS)

S3 Data (XLS)

S4 Data (XLS)

S5 Data (XLS)

S6 Data (XLS)

S7 Data (XLS)

S8 Data (XLS)

S9 Data (XLS)

S10 Data (XLS)

S11 Data (XLS)

S12 Data (XLS)

S13 Data (XLS)

S14 Data (XLS)

S15 Data (XLS)

S16 Data (XLS)

S17 Data (XLS)

S18 Data (XLS)

S19 Data (XLS)

S20 Data (XLS)

S21 Data (XLS)

S22 Data (XLS)

S23 Data (XLS)

S24 Data (XLS)

S25 Data (XLS)

S26 Data (XLS)

S27 Data (XLS)

S28 Data (XLS)

S29 Data (XLS)

S30 Data (XLS)

S31 Data (XLS)

S32 Data (XLS)

S1 Raw Images (XLSX)

S2 Raw Images (XLSX)

S3 Raw Images (XLSX)

S4 Raw Images (XLSX)

S5 Raw Images (XLSX)

S6 Raw Images (PZFX)

S7 Raw Images (PZFX)

S8 Raw Images (R)

S9 Raw Images (PNG)
