# Supplementary figures and images for: Development of electrophysiological and morphological properties of human embryonic stem cell-derived GABAergic interneurons at different times after transplantation into the mouse hippocampus
Source: PLoS One. 2020 Aug 19;15(8):e0237426. doi: 10.1371/journal.pone.0237426 (PMC7444508; doi:10.1371/journal.pone.0237426)

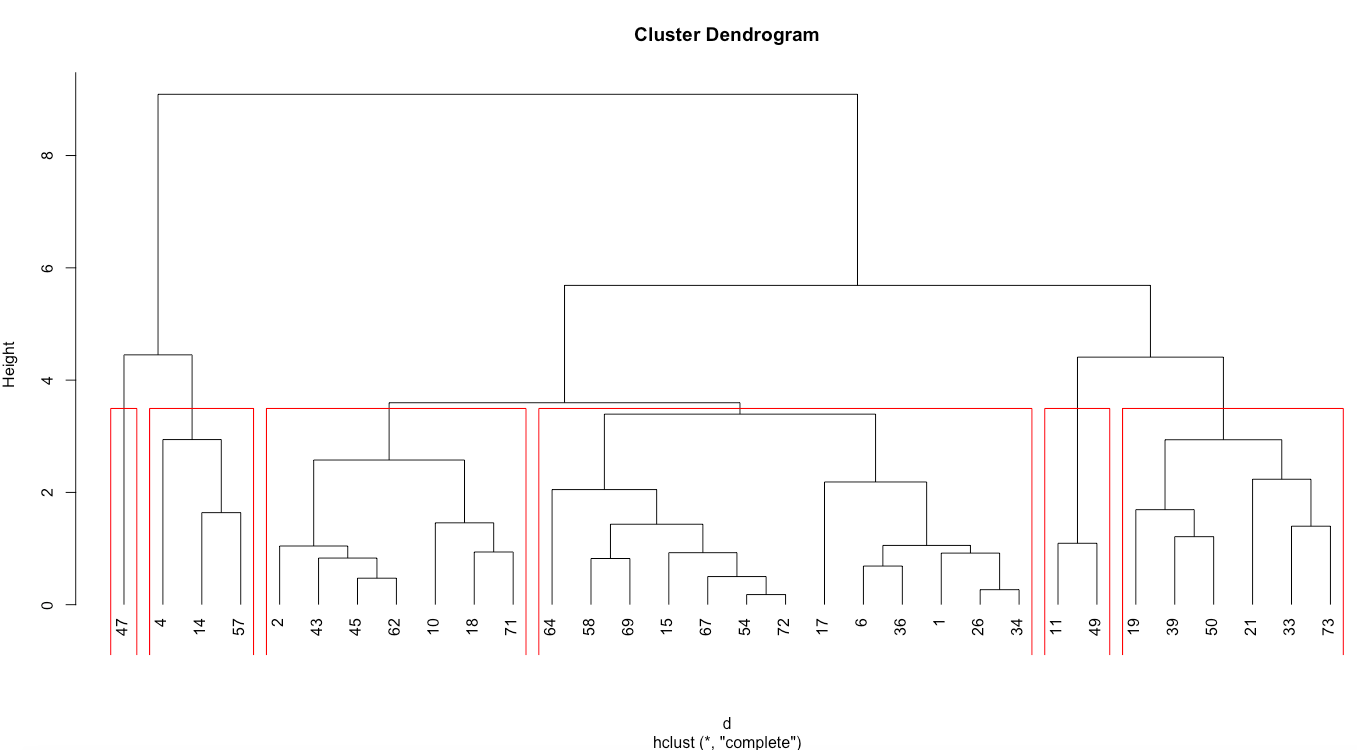

Supplement: S9 Raw Images — (PNG) [file pone.0237426.s041.png]
